# Genomic and Pathogenic Characteristics of Virulent Newcastle Disease Virus Isolated from Chicken in Live Bird Markets and Backyard Flocks in Kenya

**DOI:** 10.1155/2020/4705768

**Published:** 2020-08-18

**Authors:** Irene N. Ogali, Paul O. Okumu, Erick O. Mungube, Jacqueline K. Lichoti, Stephen Ogada, Grace K. Moraa, Bernard R. Agwanda, Sheila C. Ommeh

**Affiliations:** ^1^Institute of Biotechnology Research, Jomo Kenyatta University of Agriculture and Technology, P.O. Box 62000-00200, Nairobi, Kenya; ^2^Veterinary Science Research Institute, Kenya Agriculture and Livestock Research Organization, P.O. Box 32-00902, Kikuyu, Kenya; ^3^Department of Veterinary Pathology, Microbiology and Parasitology, University of Nairobi, P.O. Box 29053-00625, Kangemi, Nairobi, Kenya; ^4^Directorate of Veterinary Services, State Department of Livestock, Ministry of Agriculture Livestock and Fisheries, P.O. Box Private Bag, Kangemi, Kenya; ^5^Department of Zoology, National Museums of Kenya, P.O. Box 40658-00100, Nairobi, Kenya

## Abstract

Newcastle disease (ND) causes significant economic losses in the poultry industry in developing countries. In Kenya, despite rampant annual ND outbreaks, implementation of control strategies is hampered by a lack of adequate knowledge on the circulating and outbreak causing-NDV strains. This study reports the first complete genome sequences of NDV from backyard chicken in Kenya. The results showed that all three isolates are virulent, as assessed by the mean death time (MDT) and intracerebral pathogenicity index (ICPI) in specific antibody negative (SAN) embryonated eggs and 10-day-old chickens, respectively. Also, the polybasic amino acid sequence at the fusion-protein cleavage site had the motif ^112^RRQKRFV^118^. Histopathological findings in four-week-old SPF chicken challenged with the NDV isolates KE001, KE0811, and KE0698 showed multiple organ involvement at five days after infection with severe effects seen in lymphoid tissues and blood vessels. Analysis of genome sequences obtained from the three isolates showed that they were 15192 base pair (bp) in length and had genomic features consistent with other NDV strains, the functional sites within the coding sequence being highly conserved in the sequence of the three isolates. Amino acid residues and substitutions in the structural proteins of the three isolates were similar to the newly isolated Tanzanian NDV strain (Mbeya/MT15). A similarity matrix showed a high similarity of the isolates to NDV strains of class II genotype V (89–90%) and subgenotype Vd (95–97%). Phylogenetic analysis confirmed that the three isolates are closely related to NDV genotype V strains but form a distinct cluster together with NDV strains from the East African countries of Uganda and Tanzania to form the newly characterized subgenotype Vd. Our study provides the first description of the genomic and pathological characteristics of NDV of subgenotype Vd and lays a baseline in understanding the evolutionary dynamics of NDV and, in particular, Genotype V. This information will be useful in the development of specific markers for detection of viruses of genotype V and generation of genotype matched vaccines.

## 1. Introduction

Newcastle disease (ND) is a highly contagious disease of poultry that is notifiable to the World Organization for Animal Health (OIE) [1]. In developing countries, ND has a significant economic impact on the poultry sector due to the high morbidity and mortality [2]. Newcastle disease is caused by Newcastle disease virus (NDV), a single-stranded negative-sense RNA virus classified in the genus *Avulavirus* within the family Paramyxoviridae in the Order Mononegavirales [3]. Newcastle disease virus consists of a 15 kb genome with six open reading frames (ORF) encoding six major structural proteins, namely, nucleoprotein (NP), phosphoprotein (P), matrix protein (M), fusion-protein (F), hemagglutinin neuraminidase (HN), and the RNA-dependent RNA polymerase (L) in the order, 3′-NP-P-M-F-HN-L-5′ [4, 5]. Each of these genes is flanked by short extragenic “leader” and “trailer” sequences. Besides, each gene starts with a conserved gene start (GS) and ends with a conserved sequence: gene end (GE) [6].

Newcastle disease virus, although belonging to one serotype, is highly diverse genetically and antigenically. NDV isolates from the world are grouped into two distinct classes (class I or II), based on genome lengths and nucleotide sequences. Class I NDV is less divergent and belongs to a single genotype, whereas class II isolates are more diverse [5]. Based on the analysis of the complete coding region of the fusion gene, class II NDV has been divided into 18 genotypes [7–10]. Newcastle disease virus belonging to class II, which was isolated between the 1930s and 1960s, includes genotypes I, II, III, and IV. These are considered “ancient” and have genome sizes of 15,186 nucleotides (nt) [11]. The currently circulating or “recent” class II genotypes were isolated after the 1960s (V, VI, VII, VIII, and X–XVIII). The latter genotypes have an insertion of six nucleotides into the 5′ noncoding region of the nucleoprotein gene. They, therefore, possess 15192 nt in their genomes [11]. More genetic distance has been observed between “ancient” and the “recent” genotypes [5]. Some of the genotypes are further divided into subgenotypes according to set criteria [7].

The most recent genotypes (XIV–XVIII) have mostly been isolated on the African continent [8, 10, 12]. A study on isolates from Southern Africa as well as from Namibia, Zambia, Zimbabwe, and Mozambique revealed the presence of genotypes VIII and VII [13, 14]. In Zambia, genotype XIII has also been isolated from poultry [15]. A comparative analysis of APMV-1 sequences from West Africa indicated that the viruses correspond to genotypes I and VI and novel genotype XVIII [16]. Novel genotypes XIV and XVII have also been isolated from Western and Central African countries [17, 18]. Isolates from Ethiopia were assigned to genotypes VII and VI [19–21]. Isolates from Tanzania were assigned to genotypes I, V, and Via, respectively [22]. Previous studies reported that isolates from Sudan belonged to genotypes VII and VI [23, 24]. A study in Eastern Uganda in 2001 revealed that they belonged to genotype VIa , 2004) [25]. A more recent study in Uganda isolated strains of genotype Vd from poultry in LBMs [26]. It is suggested that increased surveillance on the African Continent may lead to the discovery of more genotypes and subgenotypes [27]. This is thought to be a result of the predominance of the backyard poultry management system which encourages the presence and spread of varied strains of velogenic NDV which threatens commercial poultry enterprises [28].

In Kenya, like other developing countries, the high population of poultry is kept on smallholder backyard farms. Poultry production is important for the livelihoods of many rural households as a source of protein and income and fulfills the sociocultural role. Although Newcastle disease is endemic in Kenya, potential reservoirs of NDV virus exist which are responsible for the annual epidemic outbreaks of ND causing annual epidemic outbreaks that result in high bird mortality, economic losses, and negative impact on the livelihoods of rural populations [1]. The introduction of birds from informal live bird markets (LBMs) poses an important source of introduction of NDV into a flock [29]. Live bird markets may enhance the movement of infected birds and the spread of NDV is rampant in Kenya and may be important in the epidemiology of ND [30]. However, until now, only a few studies have reported the genetic and pathological characteristics of NDV circulating in Kenya. This study for the first time characterized and compared the genomic and pathological characteristics of NDV from live bird markets and backyard poultry farms in Kenya to establish the epidemiological link and role of live bird markets and backyard poultry in the spread of NDV in Kenya.

## 2. Materials and Methods

### 2.1. Viruses

Cloacal swabs and tissue samples for the study came from chicken showing clinical symptoms of Newcastle disease. These samples were obtained from live bird markets and backyard poultry farms in Kenya during a survey of Newcastle disease virus described by [30]. We inoculated samples from individual birds in three 10-day-old specific antibody negative (SAN) embryonated chicken eggs through the allantoic cavity. Three days after inoculation, the allantoic fluid was harvested and clarified by centrifugation at 4000 × g for 20 minutes at 4°C. We used the supernatant to run the hemagglutination inhibition test using specific antisera to the reference strains of NDV. Three NDV isolates designated KE001/2015 (Makueni), KE0811/2016 (Mombasa), and KE0698/2016 (Malaba) showed high HA titer (>2^4^). The allantoic fluid of the three isolates was aliquoted for use in pathogenicity tests and genome sequencing and characterization.

### 2.2. Study Clearance and Permits of Compliance

The Kenya Agricultural and Livestock Research Organization (KALRO), Veterinary Science Research Institute- (VSRI-) Institutional Animal Care and Use Committee (IACUC), approved poultry handling procedures used in the study (protocol number: KALRO/VSRI/IACUC/1/23082017) according to the appropriate animal guidelines [31]. We carried out the study in the biosafety level II isolation facility at KALRO-VSRI.

### 2.3. Biological Characterization of the NDV Isolates

The pathogenic potential of the three NDV isolates was evaluated using a standard assay to determine mean death time (MDT) in 10-day-old specific antibody negative (SAN) embryonated eggs and the intracerebral pathogenicity index (ICPI) in 1-day-old chicks [1]. Briefly, ICPI was performed in 1-day-old chicks by inoculation of 50 *μ*L of allantoic fluid diluted 10-fold in PBS without antibiotics as recommended [1]. We calculated the intracerebral pathogenicity index as the mean of each observation for each bird. We observed the birds every 24 hours for 1 week. At each observation, the birds were scored 0 if normal, 1 if sick, and 2 if dead. We humanely sacrificed birds that were too ill and unable to eat by cervical dislocation and scored them as dead in the next observation. We gave dead birds a score of two at each of the remaining observations.

We considered Newcastle disease virus strains with ICPI score of 0.7 lentogenic, while those with a score of more than 1.5 as velogenic. On the other hand, those strains with ICPI scores between 0.7 and 1.5 were mesogenic. For MDT, allantoic fluid with virus was diluted in a series of 10-fold dilutions with phosphate-buffered saline (PBS) and each dilution inoculated into the allantoic cavity of specific pathogen-free 10-day-old embryonated eggs and incubated at 37°C. We observed the eggs every 8 hours for 7 days and the time of embryo death was recorded. The MDT was recorded as the mean time (hours) for the minimum lethal dose of the virus to kill all the embryos [1]. We considered isolates with MDT ≤60 hours velogenic: MDT between 61 and 90 hours was mesogenic, while those with MDT >90 hours were lentogenic.

### 2.4. Pathological Characteristics of the NDV Isolates

To determine the pathological characteristics of the NDV isolates, we randomly allocated forty (40) SAN chicken aged four (4) weeks into four experimental groups consisting of three NDV infected groups (group 1-KE001/2015, *n* = 10; group 2-KE0698/2016, *n* = 10; and group 3-KE0811/2016, *n* = 10) and one control group (*n* = 10). We challenged birds in the NDV infected groups via intraocular inoculation with respective virus suspension (0.1 ml) containing 10^6^ 50% lethal dose (LD50). We inoculated the control group with 0.1 ml PBS and observed all birds daily for clinical signs of disease. We euthanized and necropsied two birds from each group at day 5 after inoculation and all birds with severe clinical symptoms. We monitored the birds daily until the end of the experiment, two weeks after challenge. Thereafter, we euthanized and necropsied all remaining birds. At necropsy, we collected tissues of trachea, spleen, brain, lung, liver, bursa of Fabricius, proventriculus, and caecal tonsils and fixed them in 10% buffered formalin. The samples were embedded into the paraffin and sectioned at 3 *μ*m and routinely deparaffinized and stained with hematoxylin and eosin for histopathological examination.

### 2.5. RNA Extraction and Whole-Genome Sequencing

We extracted viral genomic RNA from the allantoic fluid using Trizol reagent (Life Technologies, USA) according to the manufacturer's instructions. The concentration and purity of extracted RNA were measured using a spectrophotometer (ND-1000, Nanodrop Technologies, USA) and Qubit Fluorimeter (Invitrogen, Carlsbad, CA, USA), and the integrity of RNA was visualized by electrophoresis in a 1.2% formaldehyde agarose gel stained with GelRed and also using the Bioanalyzer. RNA extracts were selected for library preparation when the 260/280 purity index was ≥2.0 and the integral RNA in electrophoresis and Bioanalyzer measurements was greater than eight (RIN > 8). The concentration and purity of the extracted RNA were determined using a Qubit Fluorimeter (Thermo Scientific, Walton, Massachusetts, USA) before storage at −80°C until use.

The nucleotide sequence of the full viral genome was determined using next-generation sequencing (NGS). The cDNA libraries were prepared using a TruSeq RNA sample preparation kit (Illumina, CA, USA). Briefly, mRNA was prepared by purification and fragmentation of 2 *μ*g of total RNA using oligo (dT) magnetic beads and was used as a template for cDNA synthesis by random hexamer priming. Paired-end (2 × 100 reads) sequencing was done using Miseq (Illumina). The reads of the full-length viral genome were assembled *de novo* with SPAdes assembler version 3.10.1 [32]. We confirmed the NGS results with conventional Reverse-Transcription polymerase chain reaction (RT-PCR) using virus-specific primers. This involved purification of amplified products using Qiagen PCR Purification Kit and sequencing purified products by the Sanger method at Macrogen, Korea. The sequences obtained by the Sanger method were 100% identical to the NGS sequences.

### 2.6. Phylogenetic Analysis

We obtained complete sequences of reference strains for each NDV genotype from GenBank including the recent Tanzania isolate MK583011 (Supplementary Table S1). We also compared the study isolates with other NDV isolates from Africa, which are present in the GenBank. These sequences from GenBank and the complete coding sequences of the four NDV isolates were aligned using MUSCLE v. 3.8.31 [33, 34]. Phylogenetic and molecular evolutionary analyses were conducted using MEGA (Molecular Evolutionary Genetics Analysis) version 6.0 [35]. We constructed phylogenetic trees using the Maximum Likelihood (ML) method and estimated the tree using best-fit general time-reversible (GTR) model of nucleotide substitution with gamma-distributed rate variation among sites. We employed a bootstrap resampling process (1000 replications) to assess the robustness of individual nodes of phylogeny. Evolutionary distances were computed by the Pairwise Distance method using the Maximum Composite Likelihood method [35].

## 3. Results and Discussion

### 3.1. Biological Characterization

The three isolated viruses were virulent by the OIE standard criteria.  Table 1 shows the MDT in embryonated eggs and ICPI values for the three NDV isolates as well as the amino acid sequence at the cleavage site. The ICPI for the three isolates was >1.5, while the MDT was <60 hours indicative of velogenic strains. Further, the amino acid sequence motif at the cleavage site of the fusion gene concurred with the ICPI and MDT scores of the three isolates that indicated their velogenic nature.

### 3.2. Pathological Lesions

**Figure 1:**
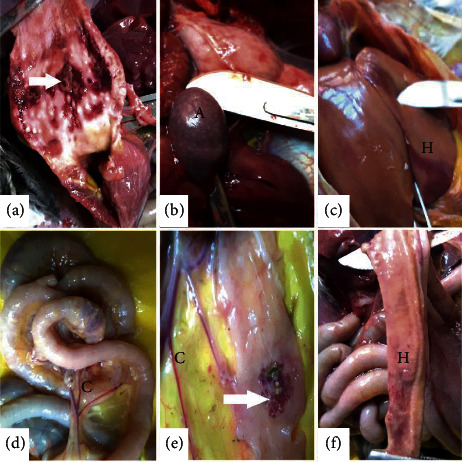
Gross pathology lesions observed on organs of specific-pathogen-free chicken infected with NDV isolates at 5 days after challenge. First row: (a) proventriculus showed patches of reddened areas on the mucosa (hemorrhagic patches), (b) the spleen was oedematous and showed multifocal white specks (necrotic points-A), and (c) the liver was oedematous and showed focal reddened hemorrhagic areas (H). Second row: ((d–f)) small intestines showed congestion of blood vessels (C), darkened patches of necrosis (arrow), and diffuse hemorrhages (H) on the mucosa.

All chickens inoculated with NDV study isolates were successfully infected and showed signs of depression, mucoid diarrhea, leg paralysis, comb cyanosis, and head edema and facial swelling with mucoid exudate from the nostrils. Gross lesions consisted of enlargement of organs such as the liver, spleen, and kidneys in the majority of the birds. Gross lesions were also evident in the mucosa of the proventriculus, spleen, liver, and small intestines (Figure 1).

These lesions included hemorrhagic and necrotic patches and blood vessel congestion. The spleen had numerous multifocal whitish spots that were evident on the surface. The trachea also showed mucosal hemorrhages and necrosis with a few birds showing pneumonic consolidation in the lungs with the discharge of caseous exudates from the air sacs. The intestines showed darkened areas of necrosis that were evident on the serosa surface. These necrotic areas in the lumen of the intestines were covered with mucoid content underneath of which were ulcerative foci. The bursa of Fabricius showed marked edema (Figure 1).

All groups infected with the NDV study isolates presented similar histological changes in the multiple organs and tissues at 5 days after challenge. The most extensive damage involved blood vessels and lymphoid tissues. We observed vascular changes including hemorrhages, congestion, and edema in majority of the organs. Severe congestion of blood vessels and diffuse hemorrhages were present in the liver, small intestines, and kidneys.

**Figure 2:**
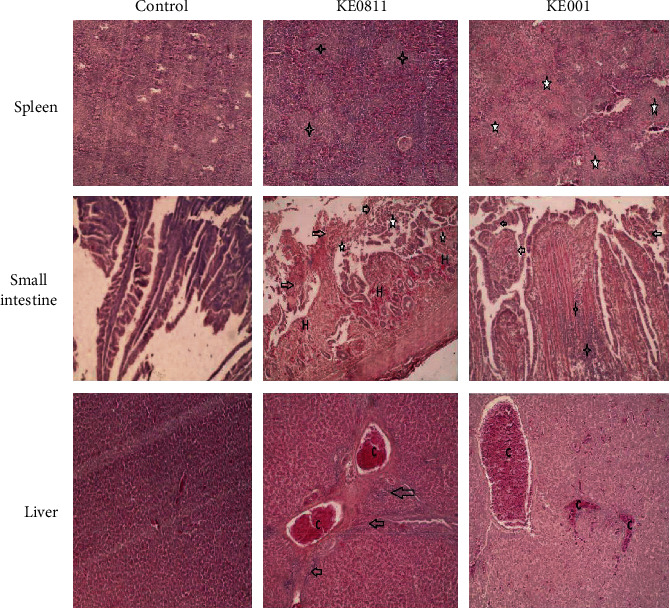
Photomicrographs showing hematoxylin and eosin (HE) staining on sections of the spleen, small intestines, and liver at 5 days after challenge. Magnification for panels of spleen and liver and 100x for small intestines × 400. First row: spleen tissue samples from isolate: KE0811 and KE001 showed lymphocyte depletion in the follicles and inflammatory exudates (star). Second row: small intestine tissue samples from NDV challenged groups showed multifocal mucosal hemorrhages (H), lymphocytic infiltration of the submucosa (star), and sloughing of mucosa (arrow). Third row: liver tissue samples of NDV challenged group showed congestion of hepatic blood vessels (C) and lymphocyte infiltration of periportal areas (arrow).

We observed depletion of the lymphoid tissue in lymphoid organs such as the spleen, bursa, and payers patches. On the other hand, organs such as lungs, small intestines, and liver showed lymphocyte infiltration (Figure 2). The intestinal mucosal epithelium was denuded with loss of villi structure. The findings resemble pathological presentation in infection by highly virulent viruses [36].

### 3.3. Genome Similarity Analysis

Similarity search of the three isolates on the nucleotide databases through the BLAST algorithm yielded the highest similarity (97%) to the genome sequence of a previous Kenyan *Newcastle disease virus* (*NDV*) isolate, *A48* (Accession *JQ217420*), and Tanzanian isolate, *Tanzania/Mbeya/MT15/2012*. The isolates also showed high similarity (92%) to the complete genome sequence *Newcastle disease virus strain*, *Largo/71* from the USA classified as genotype V clade II.

The percentage similarity for nucleotide sequences encoding the different NDV genes was compared between isolate KE001/2015 (Makueni) and other NDV strains including the study strains: KE0811/2016 (Mombasa) and KE0698/2016 (Malaba) and others from the GenBank (NCBI). Study isolates were highly similar to NDV strains grouped under genotype V ranging from 89 to 97 (Table 2). On the other hand, the similarity of the study isolates to NDV strains of genotypes I and II ranged between 82 and 83 percent. The similarity of the coding regions of KE001/2015 (Makueni) to NDV vaccine strains (*LaSota*, *BHG*, and *I*-*2*) ranged from 77 to 85%, while the similarity to gene sequences of genotype V strains was the highest ranging from 87 to 98%. We observed the lowest similarity between the study and vaccine strains in the coding sequence of the V and P gene.

### 3.4. Genomic Features of Coding and Noncoding Regions of Isolates

The genomes of the three isolates had a length of 15192 nucleotides with the insertion of six nt (cytosine: “CCCCC (T) C”) in the 5′ UTR noncoding region of the NP gene between position 1643 nt and 1650 nt. The genome of the three isolates was organized as 3′-NP-P-M-F-HN-L-5′. The genomes started with a leader and trailer sequences at the 3′ and 5′ end with lengths of 330 55 nt and 114 nt, respectively (Table 3). Similarly, the isolates had a considerably conserved 10 nt sequence at the start of every gene (GS) and another one at the end (GE) of each gene. The open reading frame of each gene was flanked with 3′ and 5′ end untranslated regions (UTRs) of varying lengths on each end (Table 3). Between GE of one gene and GS of the next gene was a conserved sequence, intergenic sequence (IGS). The length of the nucleotide and amino acid sequence of each gene was varied with the RNA-dependent RNA polymerase (L) gene presenting the longest gene.

### 3.5. Genomic Features of Protein-Coding Regions of Isolates

In multiple sequence alignment, the NP protein of the study isolates showed the highest similarity to NDV strain A48/Kenya and Mt/Tanzania. The amino acid sequence of the three isolates had three conserved domains on the NP protein: region 1 spanning between aa 171 and 181 with the motif: ^171^QVWVTVAKAMT^181^; region 2 spanning between aa 267 and277 with the motif: ^267^FFLTLKYGINT^277^; and the third conserved region between aa 322 and 336 with the motif: ^322^FAPAEYAQLYSFAMG^336^. The latter region found in the NP ORF (Open reading frame) of all paramyxoviruses of the genera *Avulavirus* is a 15 amino acid region and was similarly conserved in all genotype representative strains of NDV used in this study. These regions are important for the formation of the NP-P complexes and regulation of viral replication [37–39].

**Figure 3:**
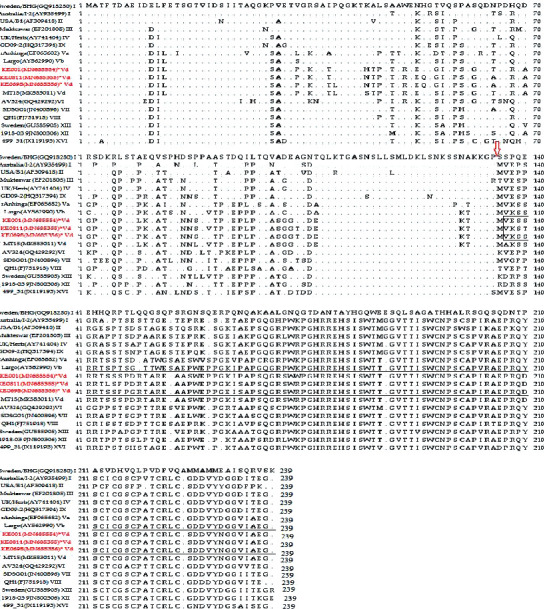
Alignment of the V protein of NDV isolates. The amino acid sequence of the V protein of the study isolates (highlighted in red) was aligned with NDV V proteins of each genotype reference strain. The P gene-editing site is indicated with an arrow.

The phosphoprotein (P) gene had the most variable sequence high variability between the three isolates and other NDV strains but had a conserved site region at nucleotide positions 394–401 (5′-AAAAAGGG-3′) corresponding to RNA editing site in the P gene [40]. At this site, insertion of one G gives rise to the V protein, while the addition of two Gs gives rise to W protein. The V protein sequence of the study isolates aligned with that of reference genotypes of NDV. The length of the V protein is 239 aa for the NDV strains (Figure 3). The V protein forms into unique folds through seven cysteine amino acids at the carboxyl- (C-) terminus. For the three isolates, the cysteines are at positions 196, 200, 212, 214, 217, 221, and 224. The V protein of the study isolates was 96.5% similar to that of Tanzanian isolate: *Mbeya/MT15/2012* (MK583011) and 90.8% similar to strain Largo/71.

The matrix protein of the three isolates had conserved sites among them: ^40^QYRIQRLDSWIDSKE^54^, ^171^YKVNFVSLTVVPRKD^185^, ^213^EVDPRSPLVKSLSRS^227^, ^247^KKGKKVTFDKLERKIRR^263^, and ^349^KIEKRHTIAKYNPFK^363^, which were similar in other NDV strains except for a few substitutions. These sites correspond to immunodominant epitopes of the matrix protein [41]. For the M protein to be localized in the nucleus, it utilizes nuclear localization signals (NLS). For the study isolates, the NLS was ^246^DKKGKKVTFDKLERKIRR^263^.

In the fusion protein sequence of the three isolates, the 5 potential asparagine (N) linked glycosylation sites, 85, 191, 366, 447, and 471, as well as cysteine residue positions, 76,199, 338, 347, 362, 370, 394, 399, 401, and 424 and individual residues, D72, E74, A75, K78, A79, and L343, were highly conserved in the study isolates. Besides, the study strains and other genotypes shared similar neutralizing antibody epitope amino acid residues of the fusion gene at positions 151 to 171, ^151^ILRLKESIAATNEAVHEVTDG^171^. The conserved sites have been reported as important functional sites of the F gene [42, 43]. The key function of the F protein is to initiate the fusion of viral surface to the host cell membrane. The amino acid motif at the F gene cleavage site is a major determinant of virulence. For the study isolates, the cleavage site had the motif ^112^R-R-Q-K-R-F^117^ corresponding to the cleavage site of velogenic viruses.

The HN protein plays a critical role in virus infectivity by binding sialic acid-containing receptors. We analyzed the HN protein of study isolates to identify the amino acid residues essential for receptor recognition. Fourteen residues essential for receptor recognition, R174, I175, D198, K236, E258, Y299, Y317, E401, R416, R498, R516, Y526, and E547, in the HN protein of the study isolates were similar to that of other NDV reference strains. To further understand the structure of the HN protein of the study isolates, the seven antigenic sites within the HN protein involved in the formation of the three-dimensional HN molecule were compared across the NDV reference strains (Table 4).

Compared to the strains I-2, we observed substitutions at different antigenic sites in the HN protein of the study isolates including D569N at antigenic site 2, N263R at site 3, R321K and K333R at site 4, and D494N at site 12. For the study isolates, the sequence at the antigenic determinant linear epitope of HN at position 345–353 had the motif ^345^PDEQDYQVR^353^ with an I/V substitution at aa 352. However, there was a substitution E347K for one study isolate, KE0698 (*MN685356*). Like other NDV strains, the HN protein of study isolates had a conserved amino acid sequence ^79^DVIDRVYKQVALESPLALLSTESIIMNAITSLSYQIN^115^ within the dominant linear antigenic domain of the HN gene which spans aa 53 to 192 [44]. This was similar in NDV strains except three substitutions at residues V81I, N98S, and T102I observed in the study isolates. The sialic acid binding site of the HN protein [45] of the study isolates, like other NDV strains, was located at aa positions 234–239. For the study isolates, the binding site had the motif ^234^NRKSCS^239^. Other amino acid residues that form HN antigen epitopes were similar in the study isolates and other NDV strains including K138, P244, F277, T380, T409, and H482. These sites correspond to the neuraminidase active site that binds the fusion protein [46].

The RNA-dependent RNA polymerase (L) gene of the study isolates and other NDV strains had a highly conserved region between aa 740 and 753 (^740^SHCRVACMVQGDNQ^753^) which corresponds to the putative active site of the L gene essential to polymerase function [47]. Other conserved domains of the L gene were observed between aa 503 and 607 and 418 between aa 634 and 854. The L gene protein was highly conserved among the NDV structural 419 proteins in the study isolates.

### 3.6. Phylogenetic Analysis

**Figure 4:**
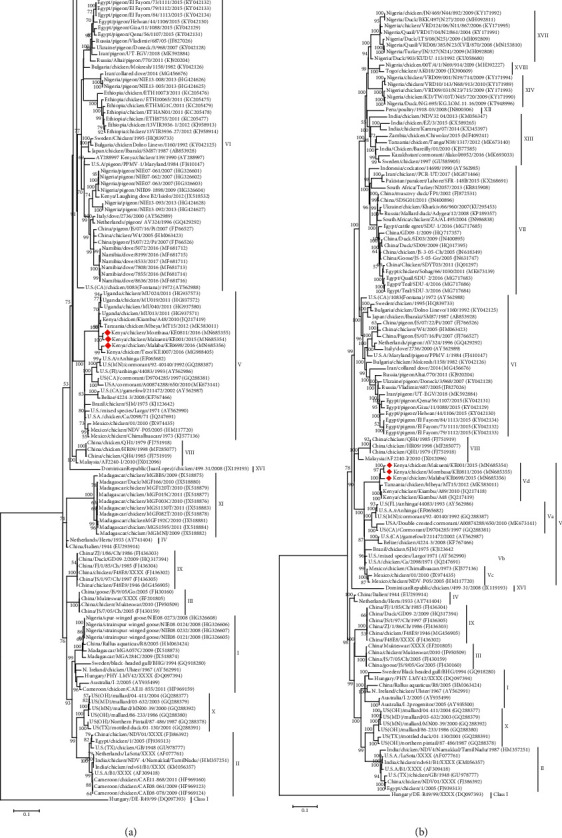
Phylogenetic analysis of NDV complete genome nucleotide sequences. The evolutionary history was inferred by using the Maximum Likelihood method. The percentage of trees in which the associated taxa clustered together based on 1000 replicates is shown next to the branches. (a) The tree was constructed based on 306 nucleotide sequences of the fusion gene with a total of 1662 positions in the final dataset. Highlighted are sequences of isolates from Kenya that were collected in the study. The tree was constructed based on the GTR model with a discrete gamma distribution used to model evolutionary rate differences among sites (2 categories (+*G*), parameter = 0.33) [35]. (b) A phylogenetic tree constructed using 122 nucleotide sequences including most known African isolates. The tree is based on the General Time Reversible (GTR) model with a discrete gamma distribution used to model evolutionary rate differences among sites (2 categories (+*G*), parameter = 0.4054). Highlighted are sequences of Kenyan isolates collected in the study.

Eighteen genotypes (I–XVIII) of NDV class II have been described [5, 17]. Phylogenetic analysis was based on the complete genome sequences including genome sequences of known strains isolated from African countries including South Africa, West Africa, and East Africa (Figure 4). The phylogenetic analysis of complete genome sequences revealed that the study isolates were clustered separately from the sequences from South Africa, which were classified as genotypes VII and XII and those of West Africa that were classified in genotypes XIV and XVIII. However, the study isolates clustered together with others from East Africa (Tanzanian and previous Kenyan strains) as well as other strains from the United States of America grouped in genotype V (Figure 4).

Within genotype V, the East African strains including study strains formed a closer cluster that has been referred to as new subgenotype Vd [5]. The genotype V of NDV is separated into four subgenotypes (Va–d). With the earlier classified subgenotypes, Va, Vb, and Vc hav been isolated mainly from Europe, Central America, and South America [9, 48, 49]. To confirm the results obtained by genome analysis, we performed a phylogenetic analysis based on the complete F gene coding sequence, which incorporated more sequences representing the known subgenotypes of genotype V (Figure 4(a)).

## 4. Conclusion

Complete genome, protein level, and biological analysis of three NDV isolates from backyard chicken in Kenya demonstrated that the isolates have the biological and pathological characteristics of virulent viruses. Further, the study demonstrated that the isolates belong to genotype V and subgenotype Vd. This is, therefore, the first genomic and pathological characterization of NDV of subgenotype Vd. Subgenotype Vd seems to be responsible for current outbreaks in Kenya and parts of the East African region. NDV isolates, sequences, and findings obtained from the study will, therefore, be valuable in analyzing the nature and diversity of NDV in the East African region. The study findings contribute to the currently available genome data on NDV in Africa and worldwide. Through this work, we also establish local and regional reference viruses for future studies in the development of improved control and diagnostic strategies.

## Figures and Tables

**Table 1:** Results of pathogenicity index tests of three NDV isolates from Kenya.

Strain	Place of origin	Accession number	MDT	ICPI	F_0_ cleavage site (111–118)
KE001/2015	Makueni	MN685354	48.7	1.56	GRRQKR_∗_FV
KE0811/2016	Mombasa	MN685355	44.2	1.69	GRRQKR_∗_FV
KE0698/2016	Malaba	MN685356	38.9	1.74	GRRQKR_∗_FV

**Table 2:** Percent (%) similarity of isolate KE001/2015 to nucleotide coding sequences of NDV reference strains.

Strain	Genome	Coding regions						
		NP	P	V	M	F	HN	L
KE0811/2016 (Mombasa)	98.94	97.34	98.82	96.19	98.99	96.49	96.85	99.06
KE0698/2016 (Malaba)	96.63	96.8	96.54	95.87	97.35	96.18	96.73	97.0
A48-Vd	95.61	97.82	—	—	97.44	97.21	97.66	97.64
Mt15/Tanzania-Vd	96.76	97.75	96.46	96.50	96.80	96.61	97.02	97.56
Largo/71-*Vb*	92.25	93.06	90.65	90.79	91.87	91.89	92.13	93.87
rAnhinga-Va	89.15	90.27	87.20	87.61	91.87	89.89	89.45	91.44
Italien-IV	86.28	88.29	84.34	83.80	86.21	87.47	87.0	88.72
QH1-VIII	86.49	88.23	85.10	84.12	84.01	86.02	85.95	88.90
AV324/96-VI	85.53	87.68	82.82	81.90	86.48	86.87	86.71	87.73
Mukteswar-III	84.79	85.23	81.81	82.22	85.11	85.84	85.02	87.52
ZJ/1/86/Ch-IX	84.58	86.73	82.40	81.26	84.65	85.60	84.79	87.64
Mali/ML007/08-XIV	84.50	85.98	83.41	80.89	84.56	84.87	84.79	87.21
ZA/AL495/04-VII	84.22	85.91	81.39	83.43	85.75	85.48	86.07	87.34
499-31/2008-XVI	83.36	86.19	80.55	81.26	84.47	83.0	84.32	85.97
MG/1992/08-XI	82.78	84.28	80.97	78.02	83.37	82.87	83.04	84.84
I-2-I	82.69	83.87	78.70	79.04	83.28	83.0	82.98	85.48
BHG-I	82.32	83.46	78.70	79.68	81.73	83.24	82.69	85.30
LaSota-II	82.12	84.14	80.30	79.68	81.91	83.36	82.68	85.10

NP: nucleoprotein; P: phosphoprotein; F: fusion protein; M: matrix protein; HN: hemagglutinin neuraminidase protein; L: RNA-dependent RNA polymerase.

**Table 3:** Genomic features of three NDV isolates from backyard chicken flocks in Kenya.

ORF	Genome characteristics					IGS	Gene length	Protein length
	Gene start	3UTR	Coding sequence	5UTR	Gene end			
NP	56–65	66	122–1591	216	1798–1808	1	1753	489
P	1810–1819	73	1893–3080	180	3250–3260	1	1451	395
M	3262–3271	34	3296–4390	112	4493–4502	1	1241	364
F	4504–4513	46	4550–6211	84	6285–6295	31	1792	553
HN	6327–6336	91	6418–8133	195	8318–8328	47	2002	571
L	8376–8386	11	8387–15001	77	15069–15078	NA	6703	2204
Leader	1–55	NA	NA	NA	NA	NA	55	NA
Trailer	15079–15192	NA	NA	NA	NA	NA	114	NA

NP: nucleoprotein; P: phosphoprotein; F: fusion protein; M: matrix protein; HN: hemagglutinin neuraminidase protein; L: RNA-dependent RNA polymerase; 3UTR: 3′ end untranslated regions (UTRs); 5UTR: 5′ end untranslated regions (UTRs); ORF: open reading frame; IGS: intergenic sequence; 1: coding sequence including stop codon; 2: number of amino acid bases without stop codon.

**Table 4:** Comparison of the amino acid residues at the antigenic sites of the hemagglutination neuraminidase protein of the Newcastle disease virus.

		Amino acid residues at different antigenic sites																	
		1	2				3		4			12		14			23		
		345	513	514	521	569	263	287	321	333	356	494	516	347	350	353	193	194	201
*Virus Strain (Accession)*	Genotype
I2 (AY935499)	I	P	R	I	S	D	N	D	R	K	K	D	R	E	Y	R	L	S	H
Lasota (AF077761)	II								K			G							
Mukteswar (EF201805)	III					G	K		K	R									
Herts (AY741404)	IV						S		K	R									
MT15 (Mk583011)	Vd					N	R		K			N							
KE001 (MN685354)	SS					N	R		K	R		N							
KE0811 (MN685355)	SS					N	R		K	R		N							
KE0698 (MN685356)	SS					N	R		K	R		N		K					
Largo (AY562990)	Vb					N	K		R	R		N							
AV324 (GQ429292)	VI			V			K		K					G					
QH1 (FJ751918)	VIII			M			K		D	R									
SDSG01 (JN400896)	VII			V			K		K										
GU585905	XIII			V		A	K		K				Q						
1918-03 (JN800306)	XII			V			K		K			G							

HN antigenic sites 1, 2, 3, 4, 12, 14, and 23 shown on the table are essential for receptor recognition. In the table are amino acid residue substitutions at different amino acid positions at the antigenic sites on the sequence of different Newcastle disease virus strains/genotypes compared to that of strain I-2 (AY935499). The amino acid residues shown are at position 345 of antigenic site 1; amino acid at positions 513, 514, 521, and 569 at antigenic site 2; amino acid at positions 263 and 287 at antigenic site 3; amino acid at positions 321, 333, and 356 at antigenic site 4; amino acid at positions 494 and 516 at antigenic site 12; amino acid at positions 347, 350, and 353 at antigenic site 14; amino acid at positions 193, 194, and 201 at antigenic site 23. The universal one-letter codes of the amino acid residues shown are interpreted as follows: P: proline; R: arginine; I: isoleucine; S: serine; D: aspartic acid; N: asparagine; K: lysine; E: glutamic acid; Y: tyrosine; L: leucine; H: histidine; V: valine; W: tryptophan; G: glycine; A: alanine; Q: glutamine. A blank at the residues shows that the sequence of the virus strain has an amino acid similar to that of I-2 Newcastle disease virus strain at that site.

## Data Availability

The full-genome sequence of the NDV isolates analyzed in this study has been submitted to GenBank under Accession nos MN685354, MN685355, and MN685356.

## Supplementary Materials

Supplementary Materials Table S1 describes the accession numbers, strain name, year, and country of isolation of reference sequences used for comparison with study sequences. The references are of known genotypes of Newcastle disease virus and are available in the GenBank including those isolated from Africa. Figure S2 shows the whole phylogenetic tree representing full coding sequences of the fusion gene of 306 strains of NDV available in the GenBank including sequences obtained from this study and all known isolates from Africa. The fusion gene is useful in the characterization of NDV. Therefore, this Maximum Likelihood tree is useful for comparison with the complete genome phylogenetic tree that is presented in Figure 4. The two trees are similar in their characterization of study isolates.
