# Supplementary material for: Genomic and Pathogenic Characteristics of Virulent Newcastle Disease Virus Isolated from Chicken in Live Bird Markets and Backyard Flocks in Kenya
Source: Int J Microbiol. 2020 Aug 18;2020:4705768. doi: 10.1155/2020/4705768 (PMC7450340; doi:10.1155/2020/4705768)
Supplement: Supplementary Materials — Table S1 describes the accession numbers, strain name, year, and country of isolation of reference sequences used for comparison with study sequences. The references are of known genotypes of Newcastle disease virus and are available in the GenBank including those isolated from Africa. Figure S2 shows the whole phylogenetic tree representing full coding sequences of the fusion gene of 306 strains of NDV available in the GenBank including sequences obtained from this study and all known isolates from Africa. The fusion gene is useful in the characterization of NDV. Therefore, this Maximum Likelihood tree is useful for comparison with the complete genome phylogenetic tree that is presented in Figure 4. The two trees are similar in their characterization of study isolates. [file 4705768.f1.zip › 4705768.f1/4705768_ Supplementary_Figure_S2.pptx]

## Slide 1
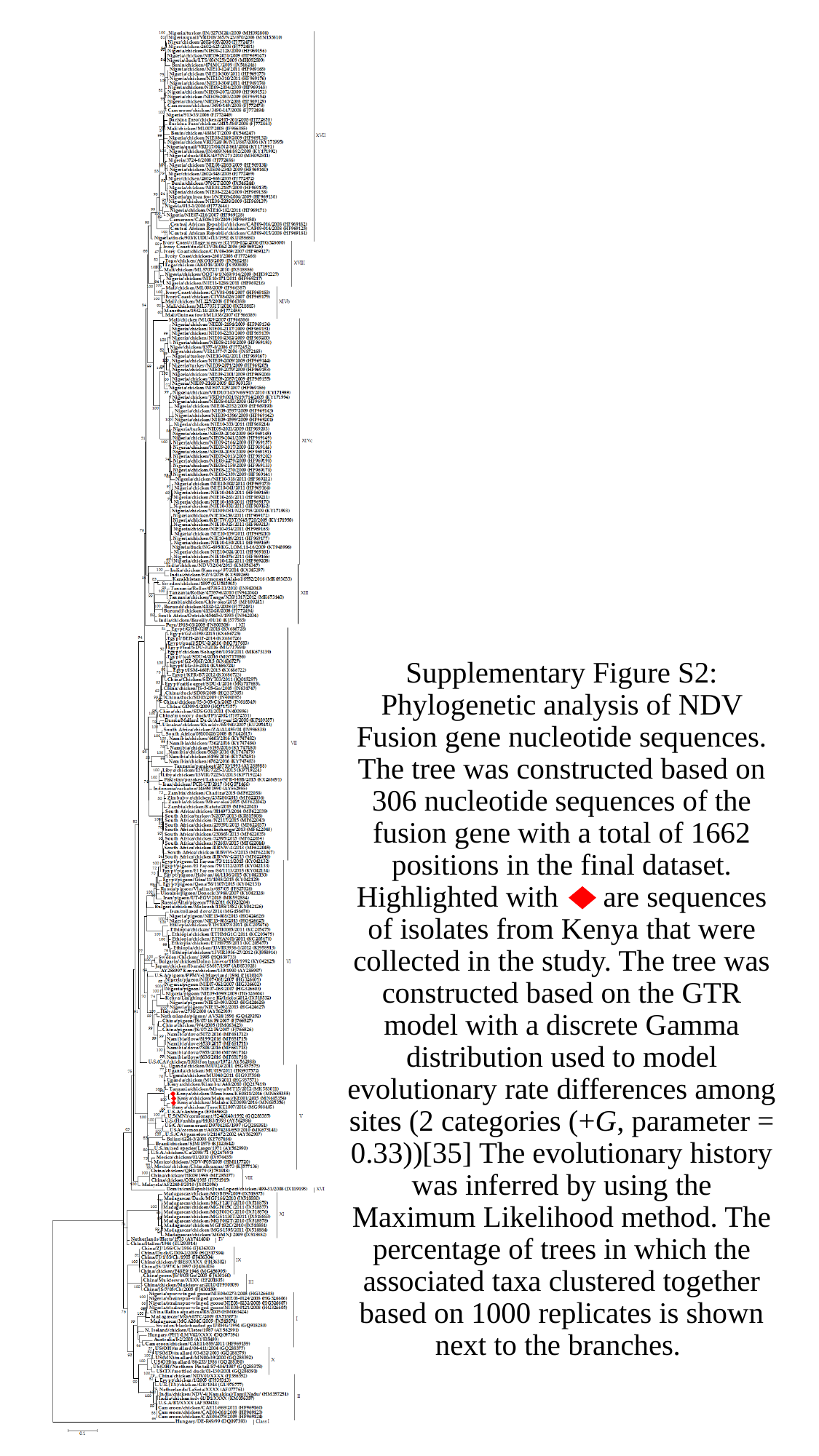

Supplementary Figure S2: Phylogenetic analysis of NDV Fusion gene nucleotide sequences. The tree was constructed based on 306 nucleotide sequences of the fusion gene with a total of 1662 positions in the final dataset. Highlighted with are sequences of isolates from Kenya that were collected in the study. The tree was constructed based on the GTR model with a discrete Gamma distribution used to model evolutionary rate differences among sites (2 categories (+G, parameter = 0.33))[35] The evolutionary history was inferred by using the Maximum Likelihood method. The percentage of trees in which the associated taxa clustered together based on 1000 replicates is shown next to the branches.
